# Community development practice in occupational therapy: A scoping review

**DOI:** 10.1111/1440-1630.70081

**Published:** 2026-03-15

**Authors:** Tetsuya Anzai, Atsushi Kawabata, Norikazu Kobayashi, Peter Bontje

**Affiliations:** ^1^ Graduate School of Human Health Science Tokyo Metropolitan University Tokyo Japan; ^2^ Research Department Tokyo Center for Dementia Care Research and Ptactice Tokyo Japan; ^3^ Graduate School of Health Sciences Hiroshima Cosmopolitan University Hiroshima Japan

**Keywords:** community development, community participation, health services, occupational therapy, professional role

## Abstract

**Introduction:**

Community development is a vital area of practice in occupational therapy, aligned with a recent shift in focus from individual‐level interventions to community and societal issues. Despite its growing significance, knowledge and understanding of community development in occupational therapy remain fragmented. This study aimed to provide a comprehensive map of how community development is researched, discussed, and conceptualised within the field of occupational therapy.

**Methods:**

A scoping review was conducted using 10 databases to identify relevant literature published up to September 2025. The search strategy combined the terms ‘occupational therapy’ with either ‘community development’ or ‘community‐centred practice’. Descriptive statistics and content analysis were used to examine the characteristics of the literature and to identify trends in the understanding of community development in the field of occupational therapy.

**Consumer and Community Involvement:**

This study did not include consumer or community involvement.

**Results:**

A total of 38 articles published between 1996 and 2025 were included, with most originating in Canada, Australia, and South Africa. Of these, 18 were empirical studies, and most employed qualitative methodologies. Findings related to community development in occupational therapy were divided into two main themes: ‘What is known about community development?’ and ‘How community development is described in relation to occupational therapy.’

**Conclusion:**

The results revealed limited contextual and methodological diversity in community development research on occupational therapy. We also identified key barriers and enablers in community development practice, offering insights to support practitioners and researchers in advancing this emerging area of occupational therapy. Future studies are required to explore community development practices in diverse geographical and cultural contexts, develop theoretical frameworks to guide occupational therapists, and apply the concept of collective occupation. Strengthening educational systems and institutional support is necessary for practitioners and students to promote and improve their engagement with community development in occupational therapy.

## PLAIN LANGUAGE SUMMARY

People's health and daily activities are shaped by many personal and social factors. Occupational therapists involved in community development (CD) focus on individual and social issues. These issues affect health and participation in meaningful daily activities. This study looked at 38 papers to see how CD is used in occupational therapy. Most of the papers came from only a few countries, and many told only the therapists' own stories. Therapists often work with community members, but they do not always get enough training, education, or help. More research is needed to help therapists effectively engage in CD. This study gives useful ideas to guide practice, but it has limits. For example, we only looked at papers written in English. We also searched with a few keywords, so some papers may have been missed.

**Key Points for Occupational Therapy**

- CD practices reflect core occupational therapy values and require context‐sensitive and critical engagement.
- Organisational and educational systems are essential to support occupational therapists in CD practice.
- Future research should strengthen theoretical foundations and include underrepresented global contexts.

## 1 INTRODUCTION

Occupational therapy has shifted from an individual‐based medical model towards greater engagement with community‐ and societal‐level issues. Furthermore, individual health is shaped by social determinants of health. Despite the long‐standing focus on individual interventions and given the critical role of social determinants in health outcomes, there is an increasing and urgent call for health professionals to broaden their scope to include societal approaches (Doll et al., 2023; Marmot et al., 2008). This paradigm shift aligns with the Sustainable Development Goals (United Nations, 2015), particularly Goals 3 and 11, which advocate health for all and particularly for the community.

Occupational participation and engagement are not only individual concerns but are shaped by social factors (Lewis & Lemieux, 2021; Laliberte Rudman, 2010; Smith, 2024). For example, the occupational justice perspective (Stadnyk et al., 2010), which provides information on how social structures affect individuals' occupational participation, guides occupational therapists on social factors that impact the participation of their clients. This growing consensus has prompted occupational therapists to engage in community‐targeted and socially targeted practices.

One type of occupational therapy that targets communities is the community development (CD) approach. CD is a broad collection of approaches that involves working in partnership with community members to prioritise and address community‐identified issues (Labonte, 1997; Lauckner et al., 2019). This concept is used primarily in Anglo‐Saxon countries such as Canada, Australia, and the United Kingdom, whereas other countries use different names or labels (Gilchrist & Taylor, 2022; International Association for Community Development, 2015). In occupational therapy, recognising the growing importance of community‐focussed interventions, the World Federation of Occupational Therapists (WFOT) has positioned CD as a key research priority and an essential component of occupational therapy education (World Federation of Occupational Therapists, 2016; World Federation of Occupational Therapists et al., 2017). This underscores the need for the profession to establish a comprehensive body of knowledge regarding CD.

Recent exploratory studies have examined the emerging practices of CD in occupational therapy (Irvine‐Brown et al., 2022; Lauckner et al., 2019; Leclair et al., 2019). These studies indicate that knowledge in this area remains fragmented and occupational therapists face challenges in articulating their approaches and maintaining clarity in practice. Conversely, Hyett et al. (2019) developed a conceptual framework of community‐centred practice (CCP), which shares core principles with CD such as working collaboratively with communities to address locally defined needs. Although the concepts overlap, the meanings of terms such as CD and CCP vary, reflecting contextual and theoretical preferences.

Additionally, review studies have examined topics related to community occupational therapy. Estrany‐Munar et al. (2021) explored the effectiveness of community occupational therapy interventions by reviewing occupational therapy research at the community level but found limited evidence of their impact. Okita et al. (2023) conducted a scoping review mapping existing occupational therapy scoping reviews to identify their characteristics, highlight research priorities, and identify the limited scope of research in this field. However, these studies focussed on intervention outcomes or provided results from narrowly targeted materials, leaving gaps in the understanding of the broader landscape of CD in occupational therapy. Furthermore, as there is a lack of CD education for occupational therapists and occupational therapy students (Lauckner, 2010; Lauckner et al., 2007; Leclair et al., 2016), a synthesis of the current knowledge and conceptualisations of CD is warranted.

Given the diverse definitions of CD (Trentham et al., 2007) and the challenges in articulating its practice, a comprehensive overview may provide valuable insights to enhance education or research on CD, and support practitioners who engage therein. Despite the growing interest therein, little is known about CD in occupational therapy. To address these gaps, this study aimed to provide a comprehensive map of how CD is researched, discussed, and conceptualised within the field of occupational therapy. This will support practitioners in adopting the best possible informed approach and contribute to the theoretical advancement of the field.

## 2 METHODS

As this study sought to map the breadth of the literature and to clarify conceptual understanding and research trends related to CD in occupational therapy, a scoping review was considered the appropriate methodology (Munn et al., 2018). The review was conducted in accordance with the relevant sections of the Preferred Reporting Items for Systematic Review and Meta‐Analysis Extension for Scoping Reviews (PRISMA‐ScR) checklist (Tricco et al., 2018). Additionally, the Arksey and O'Malley framework (Arksey & O'Malley, 2005; Levac et al., 2010) informed the data gathering and analysis using the following five key steps: (1) identifying research questions; (2) identifying relevant studies; (3) study selection; (4) charting the data; and (5) collecting, summarising, and reporting the results. The sixth optional step, consultation with stakeholders, was not included. There are no published or registered study protocols for this review.

### 2.1 Identifying the research questions

The research questions guiding this scoping review were as follows: (1) What types of studies have been conducted on CD in occupational therapy (RQ1)? And (2) how is CD in the occupational therapy field understood (RQ2)?

### 2.2 Identifying relevant studies

We focussed on two primary concepts: occupational therapy/therapists and CD. Additionally, CCP was included as an interchangeable term for CD as studies have often used these concepts synonymously (Hyett et al., 2016; Lauckner et al., 2019; Leclair et al., 2019). Based on these concepts, the following search terms were used in combination: ‘occupational therap*’ AND (‘community development’ OR ‘community‐centered’ OR ‘community‐centered’). The following criteria were applied: (a) The paper was published in English; (b) the first author was an occupational therapy or occupational science researcher; (c) the paper mentioned CD or CCP as the main topic; and (d) the paper discussed occupational therapy or occupational science concepts. Publications that did not meet the criteria were excluded. To obtain as wide a range of information as possible, there were no limitations regarding publication year. As for the types of materials, to broaden the scope of the analysis, we intentionally included dissertations and grey literature in the target documents (Arksey & O'Malley, 2005; Brown et al., 2025; Pedersen & Tingleff, 2024; Peters et al., 2021), focusing on the following materials: research papers, opinion pieces/editorials, book chapters, conference abstracts, and dissertations.

The following electronic databases were searched, as they are relevant to occupational therapy and occupational science literature and were available at the authors' affiliated institutions: PubMed, Web of Science, CINAHL Complete, Academic Search Complete, SocINDEX, the Cochrane Database, Ovid, ScienceDirect, Wiley Online Library, and ProQuest. Although a substantial number of occupational therapy journals are indexed in Scopus, this database was not used due to restrictions imposed by the authors' affiliated institutions. The final search was conducted in September 2025. Additionally, a supplementary manual search was performed using the reference lists of the included articles. A detailed search strategy for each electronic database is presented in Table 1.

**TABLE 1:** Full search strategy per database.

Data bases	Search strategies	Search field	Final search date	Records identified
PubMed	(‘occupational therapy’ [MeSH Terms] OR ‘occupational therap*’ [All Fields]) AND (‘community development’ [All Fields] OR ‘community centred’ [All Fields] OR ‘community centered’ [All Fields])	All field	2025/09/14	55
Web of Science	‘occupational therap*’ AND (‘community development’ OR ‘community centred’ OR ‘community centered’)	All text field	2025/09/14	470
CHINAHL	‘Occupational therap*’ AND (‘community development’ OR ‘community centred’ OR ‘community centered’)	All text field	2025/09/14	663
Academic Search Complete (EBSCOhost)	‘Occupational therap*’ AND (‘community development’ OR ‘community centred’ OR ‘community centered’)	All text field	2025/09/14	768
SocINDEX (EBSCOhost)	‘Occupational therap*’ AND (‘community development’ OR ‘community centred’ OR ‘community centered’)	All text field	2025/09/14	237
Cochrane Database	#1 occupational therap* #2 ‘community development’ #3 ‘community centred’ **#4 #1 AND (#2 OR #3)**	All text field	2025/09/14	5
Ovid	#1 occupational therap* .af #2 community development .af #3 community centred .af #4 community centered .af #5 2 or 3 or 4 #6 **1 and 5**	All field	2025/09/14	654
ScienceDirect	(‘occupational therapy’ OR ‘occupational therapists’) AND (‘community development’ OR ‘community centred’ OR ‘community centered’)	All field	2025/09/14	769
Wiley Online Library	(‘occupational therapy’ OR ‘occupational therapist’) AND (‘community development’ OR ‘community centred’ OR ‘community centered’)	all field	2025/09/14	475
ProQuest	(‘occupational therapy’ OR ‘occupational therapist’) AND (‘community development’ OR ‘community centred’ OR ‘community centered’)	All field	2025/09/17	800

### 2.3 Study selection

All titles retrieved from the database searches were screened for relevance, and irrelevant titles were removed. The first and second authors independently reviewed the abstracts to determine whether they met the inclusion criteria. Disagreements regarding the inclusion or exclusion of abstracts were resolved through consensus or by consulting the third and fourth authors. Full texts corresponding to the remaining abstracts were obtained and independently assessed to determine final eligibility. In cases of disagreement, the third and fourth authors were consulted.

### 2.4 Charting the data

Based on the research questions, a data extraction form was developed in Microsoft Excel. Data were collected for the following components: (a) the authors of the article, (b) year of publication, (c) the country of the first author's institution, (d) focussed concepts (CD, CCP, both concepts), (e) type of material, and (f) explanation of CD. In the case of research papers, the following data were also collected: (g) research aim, (h) research design, (i) country where the research was conducted, (j) participants, (k) data materials for analysis, (l) number of participants, and (m) key findings. The data were charted by the first author. Subsequently, all charted data were independently reviewed by the second author to ensure accuracy (Pollock et al., 2023). In cases of disagreement, the third and fourth authors were consulted.

### 2.5 Collating, summarising, and reporting the results

As proposed by Levac et al. (2010), we followed a three‐step process: data analysis, reporting the results, and applying meaning to the results.

#### 2.5.1 Analysing the data

We performed a descriptive numerical summary of the charted data, including Items a–e, as described. For the first research question (RQ1), other charted data were summarised, including Items g–m. The charted data for (m) key findings were classified based on their shared content. For the second research question (RQ2), using charted data Item (f) explanation of CD, a basic content analysis was employed to identify the key themes related to the description of CD (Levac et al., 2010; Pollock et al., 2023). An inductive approach (Elo & Kyngäs, 2008; Juvani et al., 2005) was applied to the content analysis because of the extensive nature of explanations and descriptions of the concepts. This approach allowed us to develop a typology and analyse the data inductively rather than forcing them into a pre‐existing framework. The analysis was conducted according to the steps outlined by Juvani et al. (2005). First, text quotes that referred to CD descriptions were charted from each material (Item f). The charted descriptions of the CD were then coded and combined into subthemes. The data were further extracted by combining subthemes with similar content into the main themes describing CD. The first author conducted the initial coding and categorisation, after which all the authors reviewed and discussed the classifications at each stage. The final themes and subthemes were determined through consensus among all the researchers to ensure trustworthiness (Elo et al., 2014).

#### 2.5.2 Reporting results

The results were organised into three sections. The first section presents an outline of all materials included in this review. The second section describes the characteristics of the research on CD in occupational therapy and addresses RQ1. The third section presents the themes identified through qualitative analysis, highlighting the key topics related to CD as discussed or explained within occupational therapy and answering RQ2.

#### 2.5.3 Applying meaning to the results

We then considered the interpretation of the results in the discussion.

### 2.6 Positionality statement

All authors are occupational therapists with doctoral degrees or are currently undertaking doctoral studies. The first and second authors, who served as primary data analysts, were doctoral students in occupational therapy affiliated with a professional organisation within a department that supports community practice of occupational therapists. They acknowledged a bias towards a better understanding of the importance of community practices, such as CD, in occupational therapy. The third author is an occupational therapist with a doctoral degree, whose research focuses on older adults and community settings. The fourth author is an occupational therapist with a doctoral degree who specialises in occupational science and has extensive experience in qualitative research.

## 3 RESULTS

### 3.1 Search results

The search identified 4896 articles from the following online databases: PubMed (n = 55), Web of Science (n = 470), CINAHL (n = 663), Academic Search Complete (n = 768), SocINDX (n = 237), Cochrane Database (n = 5), Ovid (n = 654), ScienceDirect (n = 769), Wiley Online Library (n = 475), and ProQuest (n = 800) (Table 1). After removing duplicates, 4011 articles were retained for title and abstract screening.

Screening was completed using Google Spreadsheets, resulting in 3737 excluded studies and 273 remaining studies for full‐text review. After reviewing the full text, 31 articles remained, and 242 articles were excluded for the following reasons: Not in English (n = 9), the first author was not an occupational therapist/occupational therapy student researcher (n = 110), did not mention CD or CD/CCP (n = 117), and did not focus on occupational therapy/science concepts (n = 6).

**FIGURE 1:**
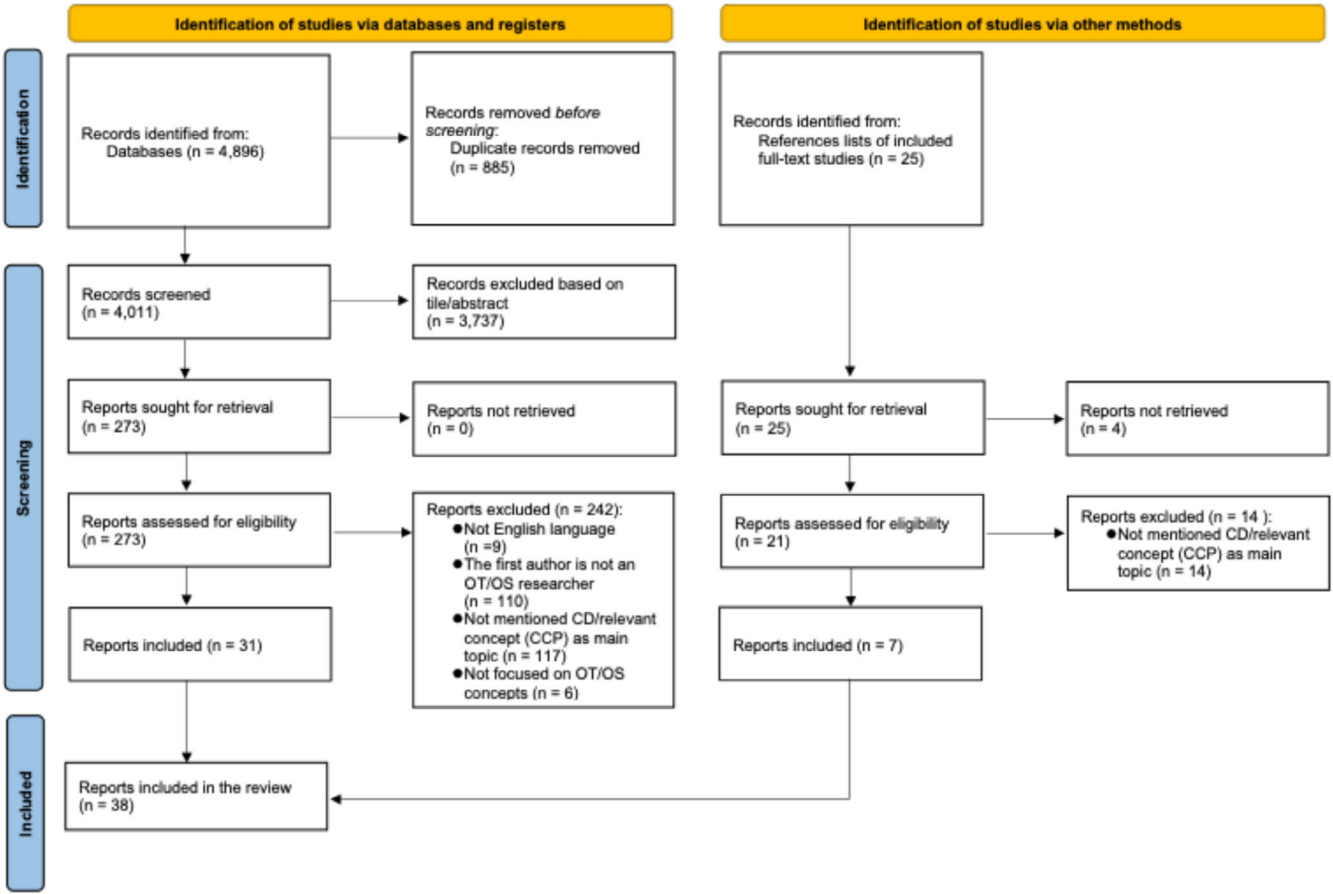
PRISMA flow diagram.

Additionally, we identified 25 articles from the reference lists of the included 31 articles, but four of these articles could not be accessed through our library services; thus, 21 remained for review. Through the same review process of screening titles, abstracts, and full texts, seven additional articles were identified in addition to the 31 previously identified articles. In total, 38 articles were included in the review. The PRISMA diagram is shown in Figure 1.

### 3.2 Descriptive characteristics

The literature selection process included 38 articles that were published between 1996 and 2025. Most of the articles originated from Canada (n = 17), followed by Australia (n = 8), South Africa (n = 6), the United Kingdom (n = 3), Germany (n = 2), France (n = 1), and New Zealand (n = 1). These articles included research papers (n = 18), opinion papers/editorials (n = 12), book chapters (n = 5), dissertations (n = 2), and others (n = 1). The primary concepts were predominantly CD (n = 32), followed by CCP (n = 4) and a combination of both (n = 2). All identified articles are listed in Table 2.

**TABLE 2:** List of identified articles (n = 38).

No.	Authors	Year	Title	Journal/book	Country	Type of material	Focussed concept
1	Westmorland	1996	The challenge of developing a community partnership model to aid the employment of people with disabilities: The Hamilton Ontario experience	Occupational Therapy International	Canada	Opinion paper/editorial	CD
2	Wilcock	1998	Occupational Therapy and public health	In A. A. Wilcock (Ed.), An occupational perspective of health (1st ed., pp. 237–251)	Australia	Book chapter	CD
3	Scaletti	1999	A community development role for occupational therapists working with children, adolescents and their families: A mental health perspective	Australian Occupational Therapy Journal	New Zealand	Opinion paper/editorial	CD
4	Banks & Head	2004	National perspective. Partnering occupational therapy and community development	Canadian Journal of Occupational Therapy	Canada	Opinion paper/editorial	CD
5	Restall et al.	2005	Inclusiveness through community development	Occupational Therapy Now	Canada	Opinion paper/editorial	CD
6	Wilcock	2006	Occupation‐focused eco‐sustainable community development approach	In A. A. Wilcock (Ed.), An occupational perspective of health (2nd ed., pp. 237–251)	Australia	Book chapter	CD
7	Lauckner et al.	2007	Exploring Canadian occupational therapists' understanding of and experiences in community development	Canadian Journal of Community Mental Health	Canada	Research paper	CD
8	Trentham et al.	2007	Health promotion and community development: An application of occupational therapy in primary health care	Canadian Journal of Community Mental Health	Canada	Opinion paper/editorial	CD
9	Townsend et al.	2007	Enabling social change	In E.A. Townsend & H.J. Polatajko (Eds.), Enabling occupation II: Advancing an occupational therapy vision of health, wellbeing & justice through occupation (pp. 153–171)	Canada	Book chapter	CD
10	Leclair	2010	Re‐examining concepts of occupation and occupation‐based models: Occupational therapy and community development	Canadian Journal of Occupational Therapy	Canada	Opinion paper/editorial	CD
11	Lauckner	2010	Conceptualizing community development from an occupational therapy perspective: Three Canadian case studies	Dissertation	Canada	Dissertation	CD
12	Lauckner et al.	2011	Conceptualizing community development: occupational therapy practice at the intersection of health services and community	Canadian Journal of Occupational Therapy	Canada	Research paper	CD
13	Windley	2011	Community development	In Role Emerging Occupational Therapy (pp. 123–134)	United Kingdom	Book chapter	CD
14	Lauckner et al.	2012	Using constructivist case study methodology to understand community development processes: Proposed methodological questions to guide the research process	The Qualitative Report	Canada	Opinion paper/editorial	CD
15	Boudreau & Donnelly	2013	The community development progress and evaluation tool: Assessing community development fieldwork	Canadian Journal of Occupational Therapy	Canada	Opinion paper/editorial	CD
16	Lauckner & Stadnyk	2014	Examining an occupational perspective in a rural Canadian age‐friendly consultation process	Australian Occupational Therapy Journal	Canada	Research paper	CD
17	Vermeulen	2015	Students' fieldwork experiences of using community entry skills within community development	South African Journal of Occupational Therapy	South Africa	Research paper	CD
18	Wilcock	2015	Occupation, environment, and community development	In A. A. Wilcock (Ed.), An occupational perspective of health (3rd ed., pp. 369–389)	Australia	Book chapter	CD
19	Leclair et al.	2016	Preparing for community development practice: A Delphi study of Canadian occupational therapists	Canadian Journal of Occupational Therapy	Canada	Research paper	CD
20	Hyett et al.	2016	Community‐centred practice: Occupational therapists improving the health and wellbeing of populations	Australian Occupational Therapy Journal	Australia	Opinion paper/editorial	CCP
21	Hyett	2016	Exploration of international case studies on community participation and health	Dissertation	Canada	Dissertation	CD
22	Mthembu et al.	2017	Spirituality in the occupational therapy community fieldwork process: A qualitative study in the South African context	Scandinavian Journal of Occupational Therapy	South Africa	Research paper	CD
23	Hyett et al.	2017	Approaches for building community participation: A qualitative case study of Canadian food security programs	Canadian Journal of Occupational Therapy	Canada	Research paper	CCP
24	Richards & Galvaan	2018	Developing a socially transformative focus in Occupational Therapy: insights from South African practice	South African Journal of Occupational Therapy	South Africa	Research paper	CD
25	Zinkstok et al.	2018	Community‐Development‐Framework	–	Germany	Other	CD
26	Janse van Rensburg	2018	A framework for occupational enablement to facilitate social change in community practice	Canadian Journal of Occupational Therapy	South Africa	Opinion paper/editorial	CD
27	Leclair et al.	2019	An occupational therapy community development practice process	Canadian Journal of Occupational Therapy	Canada	Research paper	CD and CCP
28	Lauckner et al.	2019	Moving beyond the individual: Occupational therapists' multi‐layered work with communities	British Journal of Occupational Therapy	Canada	Research paper	CD and CCP
29	Hyett et al.	2019	Re‐imagining occupational therapy clients as communities: Presenting the community‐centred practice framework	Scandinavian Journal of Occupational Therapy	Australia	Research paper	CCP
30	Carra et al.	2019	Strengthening occupational therapy practice with communities after traumatic events	British Journal of Occupational Therapy	Australia	Opinion paper/editorial	CCP
31	Irvine‐Brown et al.	2021	Re‐engaging in our role with communities: The coupling of occupational therapy and community development	Australian Occupational Therapy Journal	Australia	Research paper	CD
32	Mthembu	2021	A commentary of occupational justice and occupation‐based community development frameworks for social transformation: The Marikana Event	South African Journal of Occupational Therapy	South Africa	Opinion paper/editorial	CD
33	Irvine‐Brown et al.	2022	Exploring the Praxis of Occupational Therapy‐Community Development Practitioners	Canadian Journal of Occupational Therapy	Australia	Research paper	CD
34	Galvaan et al.	2022	Pedagogies within occupational therapy curriculum: Centring a decolonial praxis in community development practice	Brazilian Journal of Occupational Therapy	South Africa	Research paper	CD
35	Albuquerque & Farias	2022	Occupational therapists' perceptions of the need to enact health promotion in community development through occupational justice	Brazilian Journal of Occupational Therapy	France	Research paper	CD
36	Melville et al.	2023	Perceptions of occupational therapists in the United Kingdom on the applicability of the reflective framework for community development in occupational therapy	British Journal of Occupational Therapy	United Kingdom	Research paper	CD
37	Cowen et al.	2024	The role of occupational therapy in community development to combat social isolation and loneliness	British Journal of Occupational Therapy	United Kingdom	Research paper	CD
38	Hoerder et al.	2025	Justice Becomes Our Agency. Occupational Therapists in Germany Reflect on Their Practice Processes in and with Communities	Occupational Therapy in Health Care	Germany	Research paper	CD

### 3.3 Characteristics of studies on CD (RQ1)

Among the 38 articles identified, 18 were research articles. Most were qualitative studies (with one employing the Delphi method) and varied in their aims, designs, and participants or data sources (Table 3). In this section, the characteristics of the 18 research articles are summarised to address RQ1.

**TABLE 3:** List of identified research articles (n = 18).

No.	Authors and year	Research aim	Research design	Country where research was conducted	Participants (n)	Data materials	Key findings	Knowledge contribution
7	Lauckner et al. (2007)	To explore the experiences of occupational therapists in Canada working in community development, including how they understand community development and how they designed their role in this field	Qualitative study (phenomenology)	Canada	Occupational therapists (n = 12)	Transcript of interviews	The four main qualitative themes pertinent to the participants' understanding of community development and how they developed their role in this field emerged primarily through the key informant and in‐depth interviews	1
12	Lauckner et al. (2011)	To examined three cases of Canadian occupational therapists working in CD to conceptualise CD from an occupational therapy perspective	Qualitative study (multiple case study design)	Canada	Occupational therapists (n = 3) Managers (n = 4) Rehabilitation centre colleagues (n = 7) Health colleagues (n = 9) Community partners (n = 9) Service users (n = 10)	Transcript of interviews Documents Memos	A conceptual framework was developed that describes the contextual background to CD initiatives and the strategies used: nurturing community partnerships, building community capacity, influencing health services, and linking sectors	1, 3
16	Lauckner and Stadnyk (2014)	To explore the congruence between the age‐friendly community consultation process and occupational therapy practice at the community level and the lessons that can be learned from this consultation process to strengthen the applicability of the CPPF to communities	Qualitative study (reflective analysis)	Canada	Older people living in the community (n = 35) Key informants (n = 20)	Unclear	The similarities between the age‐friendly consultation and occupational therapy practice process were shown. In addition, the occupational therapists' role and using skills that enable occupation in the consultation were shown.	1, 2
17	Vermeulen (2015)	To explore the experiences of final year occupational therapy students using community entry skills during community fieldwork practice	Qualitative study (auto‐ethnographic approach)	South Africa	Occupational therapists (n = 5)	Students' own daily fieldwork reflective journals A personal narrative written by each student Transcript of a focus group discussion	Three main themes emerged from the study that tells the story of the students' experiences of using community entry skills in community fieldwork practice. These themes are (1) challenges faced in community fieldwork practice, (2) understanding culture, and (3) using community Entry skills.	1, 2, 4
19	Leclair et al. (2016)	To gain consensus among occupational therapists with expertise in community development on the knowledge, skills	Consensus method (Delphi technique)	Canada	Occupational therapists (n = 34)	Responses to questionnaires for the Delphi study	The competencies required for CD practice were identified through the tree round. The results of the Delphi highlight several areas in which occupational therapists require preparation for practice in community development. Although many of the areas identified were specific to community development practice, others were relevant to all areas of occupational therapy.	2
22	Mthembu et al. (2017)	To explore occupational therapy educators' and students' perceptions regarding spirituality in the community fieldwork practice	Qualitative study (interpretive exploratory descriptive qualitative approach)	South Africa	Occupational therapy educators (n = 9) Occupation therapy students (n = 29)	Transcript of the interviews	The evidence from this study indicated that both self‐reflection and critical reflection, using journaling, facilitated students' self‐awareness and learning about spirituality as part of the community fieldwork process. Community entry, needs identification, and ABCD are strategies of the locality development approach which emerged as enablers of spirituality in community settings.	2
23	Hyett et al. (2017)	To improve occupational therapists' understanding of an approach to building community participation, through case study of a network of Canadian food security programs	Qualitative study (qualitative case study)	Canada	Key informants (n = 5)	Transcript of interviews Field notes from field observations Public documents Webpages Social media	The four themes describe processes used for building community participation. The themes are (1) use of multiple methods, (2) good leaders are fundamental, (3) growing participation via social media, and (4) leveraging outcomes.	2
24	Richards and Galvaan (2018)	To answer the following research question: What insights could be learned from the way that occupational therapy addresses the social determinants of health through the lens of occupation‐based community development practice?	Qualitative study (autobiographical self‐study)	South Africa	Occupational therapists (n = 1) (First author)	Reflective journal Comments to occupational therapy students by the participant	The identified theme: Towards socially responsive practice revealed the value of adopting a critical and collaborative approach to practising occupational therapy in various domains of practice. Working towards social transformation was demonstrated as possible through the categories of being able to See and Feel the invisible and enabled as Equals working for change.	1, 2
27	Leclair et al. (2019)	To describe occupational therapists' practice processes when engaged in CD and the approaches occupational therapists used throughout the CD practice process	Qualitative study (interpretive description)	Canada	Occupational therapists (n = 8)	Transcript of interviews	Eight occupational therapists participated in describing a process that focussed on five key elements: (1) getting to know the community, (2) getting the ball rolling/planning together, (3) building (upon) occupational opportunities, (4) revisiting the approach, and (5) striving for sustainability. These elements occurred within a practice context and frames of reference related to CD practice. The proposed process suggests that CD practice is more complex than the CPPF outlines.	1, 2, 3
28	Lauckner et al. (2019)	To describe the practice process of occupational therapists working in community development	Qualitative study (interpretive description)	Canada	Occupational therapists (n = 12)	Transcript of interviews	There was some uncertainty among participants regarding the definition of community development. Four layers of community‐centred practice were inductively derived from the data: individual, group, community of interest, and systems. The latter two touch on community development.	1, 2, 3
29	Hyett et al. (2019)	To present a conceptual framework for community‐centred practice in occupational therapy	Qualitative study (qualitative multi‐case research)	Australia, Canada	Key informants (n = 11)	Transcript of interviews Field notes from filed observations Public documents Webpage Social media	The Community‐Centred Practice Framework was developed which can be used by occupational therapists to understand and apply a community‐centred practice approach. The framework includes four stages: (1) Community Identity, (2) Community Occupations, (3) Community Resources and Barriers, and (4) Participation Enablement.	1, 2, 3
31	Irvine‐Brown et al. (2021)	To investigate what community development and occupational therapy theory practitioners are using to guide their social occupational therapy practice, with the intention that findings may provide insights into how to support occupational therapists in their practices aimed towards social justice	Qualitative study (critical dialogical approach)	Australia	Occupational therapists (n = 4)	Transcript of interviews Additional comments by participants	Findings were grouped into two themes: (a) Occupational therapy and community development – synergies and tensions, and (b) Of the profession but not in it. Findings highlighted the theoretical shortcomings of occupational therapy for community development practice, theoretical tensions between the two disciplines, and the ‘underground’ nature of occupational therapy community development practice.	1
33	Irvine‐Brown et al. (2022)	To explore whether and how Australian occupational therapy‐community development practitioners engage in critical praxis	Qualitative study (critical dialogical approach)	Australia	Occupational therapists (n = 4)	Transcript of interviews	Findings suggest occupational therapy‐community development practitioners can engage in critical praxis but need greater support in both foundational education, and in an ongoing capacity by the profession‐at‐large, to do so effectively and consistently.	1
34	Galvaan et al. (2022)	To describe the teaching and learning practices in South Africa, University of Cape Town Occupational Therapy, Community Development Practice (UCT occupational therapist CDP) curriculum, and the pedagogy informing it	Qualitative study (multiple method)	South Africa	Occupational therapists (n = 5)	Transcript of interviews Curriculum documents	The university's curriculum related to CD practice was characterised by the theme of ‘Modelling a development process in a teaching and learning alliance.’ The approach was explained as focusing on students' willingness to face uncertainty and the critical and introspective attitude required of educators.	4
35	Albuquerque and Farias (2022)	To explore the contextually situated perceptions of the needs of four occupational therapists working in health promotion and community development in France	Qualitative study (group discussions)	France	Occupational therapists (n = 4) Social worker (n = 1) Community development advocate (n = 1)	Transcript of interviews	Participants' working needs concerning the complexities of health promotion and community development, as well as the application of the existing frameworks to support occupational therapy health promotion interventions, are summarised in four overarching themes described as follows: ‘professional skills needed to enact the community's own know‐how and self‐expertise’, ‘the importance of seeing the “whole” picture and reaching out to other sectors’, ‘the need for occupational justice to understand the complexity of community development’, and ‘the need to move beyond body functions in education’.	1
36	Melville et al. (2023)	To explore the applicability of the reflective framework for community development in occupational therapy (RFCDOT) to the UK	Qualitative study (qualitative mixed methods design)	United Kingdom	Occupational therapists (n = 3)	Answers to online questionnaire Transcript of interviews	Applicability of the RFCDOT to UK practice was evident through comparing participants' projects to key aspects of community development, their use of frameworks and models, and the RFCDOT itself. Participants also suggested adjustments to the framework to enhance its applicability to a UK audience.	1
37	Cowen et al. (2024)	To illuminate good practice for combating SIL (drawing on the findings of a broader study with older men) while highlighting the need to diversify occupational therapists' roles	Qualitative Study (multi‐method including, interview and collaborative workshop)	United Kingdom	Older men (n = 12) Community organisation staff (n = 6)	Transcript of interviews	The importance of connections, creating community and therapeutic landscapes was highlighted as beneficial to community‐dwelling older men. Additionally, the lack of appropriate local spaces to connect communities through social participation was also highlighted. This suggests the need for occupational therapists to diversify their roles, moving beyond the individual and into community development, such as serving as consultants within other disciplines to ensure the constructed world is a place where people of all ages can thrive.	2
38	Hoerder et al. (2025)	To gain a better perception and conceptualisation of community practice in Germany	Qualitative study (interpretive Description)	Germany	Occupational therapists (n = 5)	Transcript of interviews	Three main themes were identified: navigating parallel processes within the larger context or system; building community connections through occupation; growing professional identity. The utilisation of occupational science concepts was essential to work successfully on a community level.	1, 2, 3

*Note*: Knowledge contribution: (1) understanding CD practices; (2) identification of skills or perspectives for CD practice; (3) conceptualisation of CD practices by occupational therapists; and (4) providing insightful information on CD education to occupational therapy students.

#### 3.3.1 Research areas

Canada was the country from which most of the research data were collected (n = 7), followed by South Africa (n = 4), Australia (n = 3), the United Kingdom (n = 2), and France and Germany (n = 1). One of the studies collected data from Australia and Canada.

#### 3.3.2 Research aims

The identified studies had several types of aims, including exploring the experiences or practice processes of occupational therapists working in CD (n = 6); reflecting on CD practices by occupational therapists using existing theories or frameworks (n = 2); developing and evaluating new conceptual frameworks to guide CD practices by occupational therapists (n = 2); investigating the experiences and perspectives of occupational therapists (as educators) or occupational therapy students regarding CD education (including field work) at universities (n = 3); gaining consensus among occupational therapists with expertise in CD (n = 1); enhancing understanding of relevant concepts such as community participation (n = 1); examining how occupational therapists address social determinants of health from a CD perspective (n = 1); highlighting the need to diversify occupational therapists' roles in CD (n = 1); and exploring the needs of occupational therapists working in health promotion and CD (n = 1).

#### 3.3.3 Research designs

The 18 studies comprised interpretive descriptions (n = 3), case studies (n = 3), critical dialogical approach (n = 2), phenomenology (n = 1), reflective analysis (n = 1), auto‐ethnographic approach (n = 1), Delphi technique (n = 1), interpretive exploratory descriptive qualitative approach (n = 1), autobiographical self‐study (n = 1), multiple methods (n = 2), and qualitative mixed methods (n = 1).

#### 3.3.4 Participants and data materials

The participants in the 18 studies included only occupational therapists (n = 10), occupational therapists and other informants (n = 3), and non‐occupational therapy informants only (n = 5). The data materials included interview transcripts only (n = 8); interview transcripts and supplementary data such as web pages, documents, or social media posts (n = 7); and alternative types of data instead of interview transcripts, such as responses to a questionnaire and reflective journals (n = 2). One study did not clearly specify the data used to support its findings, although it described data collection through focus groups and individual interviews, in which the relationship to the results remained opaque (Lauckner & Stadnyk, 2014).

#### 3.3.5 Key findings and type of knowledge contribution

The charted data for key findings were classified into four categories of knowledge contribution based on their content: (1) understanding CD practices; (2) identification of skills or perspectives for CD practice; (3) conceptualisation of CD practice by occupational therapists; and (4) providing insightful information for CD education for occupational therapist students. When data supported two or more categories, they were applied accordingly. Further details are presented in Table 3.

### 3.4 Topics of CD which discussed or explained occupational therapy (RQ2)

We aimed to understand CD in occupational therapy to address RQ2. The charted data were organised into two main themes: ‘What is known about CD?’ and ‘How CD is described in relation to occupational therapy?’ The correspondence between each theme, the identified literature, and detailed information are provided in Table 4.

**TABLE 4:** Topics of CD that were discussed or explained in occupational therapy.

Main themes	Subthemes	Additional information/codes	Article No.
What is known about CD?	Definition of CD	Included definitions	
		Definition from non‐occupational therapy filed	
		WHO (1986)	6, 7, 8, 18, 19
		Labonte (1993)	7, 11
		Labonte (1997)	5
		Labonte (2004)	10, 12, 17
		Labonte (2012)	27, 28
		Gibbon et al. (2002)	19
		Bracht et al. (1999)	19
		Hoffman and Duponte (1992)	8
		McConnell (2002)	17
		Sen (1999)	31
		Ledwith (2016)	33
		Boutilier et al. (2000)	11
		Kenny (1994)	11
		Minkler and Wallerstein (1997)	11
		Mitchell and Wright (1992)	3
		Murray (2013)	22
		Definition from Occupational therapy filed:	
		Wilcock (1998)	2, 4, 11, 15, 17
		Wilcock (2006, 2015)	6, 18
		Restall et al. (2003)	7, 11, 15
		Lauckner et al. (2007)	11, 12, 15, 17, 19, 36
	CD's general theory or approach	Rothman and Tropman's (1987) taxonomy of community development	7, 8, 9, 10, 11, 12
		Jackson's (1989) Community Development Continuum Model	9, 10, 11, 28
		WHO's (2010) Community‐Based Rehabilitation (CBR) strategy	2, 3, 8, 28
		Kretzmann and McKnight's (1993) Asset‐Based Community Development (ABCD)	4, 22
		the five health promotion strategies in the Ottawa Charter (WHO, 1986)	11
		Bracht et al.'s (1999) the Five‐Stage Community Organization Model	28
		Laverack's (2001, 2005) community empowerment framework	11
	Distinctions between CD and community‐based practice	N/A	10, 11, 19, 22, 27, 28
	Core components of CD	Community centredness	2, 5, 10,11, 19, 20, 21, 22, 25, 31, 33
		Strengthening community capacity	5, 8, 11, 17, 27, 31, 36
		Participation	11, 13, 17
		Empowerment	6, 11, 18
		Embracing social justice	13, 17
		Self‐determination	13, 17
		Equality and justice	13, 17
		Reflection	13, 18
		Collaborative working	13, 17
		Political awareness	13
		Sustainable change	13
		Community competence and capacity building	11
		Solidarity	17
	Required skills for the CD	Understanding the community	1, 3, 5, 9, 11, 17, 19, 20,21, 22, 35
		Building relationships and collaborating with the community and local stakeholders	1, 4, 7, 8, 9, 11, 25, 32, 36
		Sharing power	3, 5, 7, 9, 11, 19, 21, 31
		Empowering people	7, 11, 17, 25, 27
	Outcomes or consequences of CD	Social change	3, 8, 31, 33, 36
		Community's ownership and sense of control of lives by residents	3, 31
		Community participation	29
		Reduction in barriers to access to local services	20
		Situation where supporters are no longer needed	31
		Outcome indicators for CD remain unclear	11
How CD is described in relation to occupational therapy	Occupational therapists' perspectives and skills	Perspective of occupational justice	1, 10, 11, 25, 35, 36, 38
		Occupational perspectives	7, 8, 20, 27, 36
		Skills for enabling occupation	20
	Occupational therapists' role in CD	Supporting the proactive problem solving of the community	5, 11, 24, 25
		Demonstrating expertise and providing unique perspectives	1, 5, 19
		Demonstrating leadership	19
		Investigating community needs and developing resources	11
		Serving as a consultant	37
		Serving as facilitators	6
		Adjusting the physical environment	5
	Characteristics of occupational therapist's role in CD	Diversity of characteristics	7, 11, 20
		Flexibility to adjust the practical context	7, 10, 20
		Lack of clarity	7, 26
	Limitations in the traditional concepts and classification of occupation	Focus on the individual when focusing on or dealing with CD practice	10, 11, 12
		Need to focus on the other classification of occupation; shared occupation or collective occupation	8, 10, 27, 32, 36
	Similarities between CD and occupational therapy	Skills required	19
		Values	11
		Client‐centredness	5, 9, 10, 11, 19, 20
		Attention to justice	11, 20, 33, 36
	Interchangeable concepts with CD	Community‐centred practice	20, 27, 28
		Social occupational therapy	31, 33
		Community participation	21
	Existing occupational therapy theories or frameworks for CD	Canadian model of client‐centered enablement	4, 11, 15, 20
		Canadian practice process framework	11, 16, 27
		Canadian model of occupation performance and engagement	11, 15
		Ecological sustainability model of health	10
		Person‐environment‐occupation model	10
		Occupational science concepts	38
		Limitations in applying these theories to CD practice	10, 11, 14, 16, 27, 31
		Necessity of conceptualising CD practice by occupational therapists to guide the practice	3, 9, 23, 26, 27, 28, 30, 32, 36
		Necessity of frameworks or theories for occupational therapists specialising in CD	4, 10, 11, 19
	Barriers and enablers to engaging in CD practice	Barriers	
		(1) Occupational therapists' individual‐level issues	
		Lack of preparation, experience, or understanding of CD	7, 10, 11, 12, 19, 26, 28, 36, 37
		Having traditional authoritarian or medically oriented perspectives	9, 12, 15, 28, 31, 36
		(2) Practice system‐level issues	
		Lack of funding, time, and support for occupational therapists working on CD practice	2, 6, 7, 11, 18, 20, 28, 37
		Lack of public awareness of occupational therapists in the field of CD	11, 20
		Lack of promotion and understanding of CD practice within the occupational therapy field	11, 31
		(3) Social‐level issues	
		Pressure to provide ‘counting’ outcomes to be provided funding and to base policies	35
		(4) Academic‐level issues	
		Lack of evidence supporting the effectiveness of CD by occupational therapists	11, 27, 28, 29, 36
		Lack of research into how occupational therapists engage in CD practices and what roles, skills, and preparation are required to engage in CD practices	7, 11, 12, 17, 20, 26, 36
		Enablers	
		(1) Development of knowledge and evidence to explain CD practice by occupational therapists	
		Research and knowledge to explain occupational therapist practice in CD	10, 11, 21, 36
		Evidence to support the effectiveness of occupational therapist‐led CD practice	7, 19, 21
		Participatory research involving both occupational therapists and local stakeholders	31
		Research demonstrating the role of occupational therapists in CD practice	4, 37
		(2) Establishment of educational systems	
		Necessary to provide education for occupational therapists	2, 6, 7, 15, 19, 20, 23, 27, 31, 36, 37
		Necessary to provide education for occupational therapy students	15, 17, 19, 21, 24, 26, 35
		(3) Promotion of CD practice by occupational therapists	
		Promote CD practice by occupational therapists	7, 21
		Start CD practice by expanding and renegotiating their existing roles	21
		(4) Strengthening of organisational systems to support CD practice	
		Create a network system that allows occupational therapists who are working on CD practice to connect with each other	7, 19, 21, 31
		Establishment of policies supporting CD practice by occupational therapists	12
		Expanding support resources for CD by occupational therapists (i.e., grants for this practice)	4

*Note*: Article No. corresponds to No. for each article listed in Table 2.

#### 3.4.1 What is known about CD?

More than half of the identified articles (n = 20; 53%) addressed the definition of CD. Among these, definitions cited in more than three articles included those by the World Health Organization (1986), Labonte (1993, 1997, 2004, 2012), Wilcock (1998), Restall et al. (2003), and Lauckner et al. (2007), which reflect both occupational therapist and non‐occupational therapist perspectives. The most frequently cited occupational therapist‐based definition was that proposed by Lauckner et al. (2007): ‘a multi‐layered, community‐driven process in which relationships are developed and the community's capacity is strengthened, in order to affect social change in their community that will promote the community's access and ability to engage in occupations’ (Lauckner et al., 2007), which was referenced in six articles. In contrast, the most cited non‐occupational therapist definition was that of Labonte: ‘the process of organizing or supporting community groups in their identification of important concerns and issues and their ability to plan and implement strategies to mitigate their concerns and resolve their issues’ (Labonte, 1993, 1997, 2004, 2012), which appeared in eight articles.

Eleven articles mentioned the general theories or approaches to guide CD, such as Rothman and Tropman's (1987) taxonomy of community development (n = 6), Jackson et al.'s (1989) Community Development Continuum Model (n = 4), and the WHO's (2010) Community‐Based Rehabilitation (CBR) strategy (n = 4). Others mentioned were Kretzmann and McKnight's (1993) Asset‐Based Community Development (ABCD) (n = 2), the five health promotion strategies in the Ottawa Charter (World Health Organization, 1986) (n = 1), the Bracht et al. (1999) Five‐Stage Community Organization Model (n = 1), and Levarack's (2001, 2005) community empowerment framework (n = 1).

Six articles discussed distinctions between CD and community‐based practice. The main differences between these practices were mentioned in terms of who took the lead in addressing community issues and the roles and expectations of the occupational therapists in each practice.

Core components of CD were also highlighted. The most frequently mentioned components were community centredness (n = 11) and strengthening community capacity (n = 7). Additionally, other CD core components included participation (n = 3), empowerment (n = 3), embracing social justice (n = 2), self‐determination (n = 2), equality and justice (n = 2), reflection (n = 2), collaborative working (n = 2), political awareness (n = 1), sustainable change (n = 1), community competence and capacity building (n = 1), and solidarity (n = 1).

Several studies examined the required skills for CD. These were understanding the community (n = 11), building relationships, collaborating with the community and local stakeholders (n = 9), sharing power (n = 8), and empowering people (n = 5).

Eight studies reported the outcomes or consequences of CD. The most mentioned outcomes included social change (n = 5), followed by community ownership and a sense of control of lives by local residents (n = 2), community participation (n = 1), a reduction in barriers to access local services (n = 1), and a situation where supporters are no longer needed (n = 1). However, it was also noted that the outcome indicators for CD remain unclear (Lauckner, 2010).

#### 3.4.2 How CD is described in relation to occupational therapy

Nine articles mentioned that occupational therapist perspectives and skills could contribute to CD. The unique perspectives and skills of occupational therapists that contribute to CD were identified from the perspectives of occupational justice (n = 6), occupational perspectives (n = 5), and skills for enabling the occupation (n = 1). Occupational justice was emphasised as a guiding perspective for occupational therapists addressing community issues and engaging in CD to promote social change.

Eleven articles addressed the role of occupational therapists in CD. This included supporting the proactive problem solving of the community (n = 4), demonstrating expertise and providing unique perspectives (n = 3), demonstrating leadership (n = 1), investigating community needs and developing resources (n = 1), serving as a consultant (n = 1) or facilitator (n = 1), and adjusting the physical environment (n = 1). The characteristics of the role of occupational therapists in CD were also discussed, including diversity (n = 3), flexibility to adjust to the practical context (n = 3), and lack of clarity (n = 2).

Three articles mentioned the limitations in the traditional concepts and classification of occupation that focus on individuals when focusing on or dealing with CD practice. Five articles mentioned the need to focus on other classifications of occupations that explain the concept of occupation shared between groups and communities, such as shared occupations or collective occupations, which transcend the individual.

Several studies highlighted the similarities between CD and occupational therapy in terms of the skills required and values. In particular, the two perspectives of client‐centredness (n = 6) and attention to justice (n = 4) were emphasised in multiple articles as common values shared by CD and occupational therapists. In addition, four articles addressed several terms as interchangeable concepts with CD, including CCP (n = 3), social occupational therapy (n = 2), and community participation (n = 1).

Eleven articles mentioned existing occupational therapy theories or frameworks for CD, including the Canadian Model of Client‐Centered Enablement (Townsend et al., 2007) (n = 4), the Canadian Practice Process Framework (Davis et al., 2013) (n = 3), the Canadian Model of Occupation Performance and Engagement (Polatajko et al., 2007) (n = 2), the Ecological Sustainability Model of Health (Wilcock, 1998) (n = 1), the Person‐Environment‐Occupation Model (Law et al., 1996) (n = 1), and occupational science concepts (n = 1). However, several articles also pointed out the limitations of applying these theories to CD practices (n = 6). These limitations include an individualistic focus that may not align with the collective and community‐centred nature of CD. In addition, the necessity of conceptualising CD practice by occupational therapists to guide the practice (n = 9) and frameworks or theories for occupational therapists specialising in CD (n = 4) were highlighted in several articles.

Many articles addressed barriers and enablers to engaging in CD practice (n = 23). The identified barriers were categorised into four levels: (1) occupational therapist individual‐level issues (e.g., lack of preparation, experience, or understanding of CD, having traditional authoritarian or medically oriented perspectives) (n = 12); (2) practice system‐level issues (e.g., lack of funding, time, and support for occupational therapists working on CD practice, lack of public awareness of occupational therapists in the field of CD) (n = 9); (3) social‐level issues (e.g., pressure to provide ‘counting’ outcomes to provide funding and base policies) (n = 1); and (4) academic‐level issues (e.g., lack of evidence supporting the effectiveness of CD by occupational therapists; lack of research into how occupational therapists engage in CD practices and what roles, skills, and preparation are required to engage in CD practices) (n = 10). The identified enablers were also categorised into four categories: (1) development of knowledge and evidence to explain CD practice by occupational therapists (n = 9), (2) establishment of educational systems (e.g., education for students and occupational therapists) (n = 16), (3) promotion of CD practice by occupational therapists (n = 2), and (4) strengthening of organisational systems to support CD practice (e.g., establishing policies and funding mechanisms to support CD practice by occupational therapists) (n = 6).

## 4 DISCUSSION

We aimed to map and summarise how CD is understood and studied in the field of occupational therapy, addressing two research questions. Our findings provide a comprehensive overview of research trends, the current state of theoretical underpinnings, and the potential barriers and facilitators influencing CD practice in occupational therapy. The results suggest that occupational therapists engage with and interpret CD through distinct professional perspectives, values, and practices. However, systemic, social, and academic barriers persist, highlighting the need for strengthened theoretical frameworks, enhanced educational strategies, and greater institutional support to advance professional CD practice. We address each research question and explore its implications for research, theory, and practice:

### 4.1 Scope and trends of CD research in occupational therapy

Although the included studies offer valuable insights into the various dimensions of CD, limitations such as narrow study designs and constrained contexts suggest that this area of research remains in its early stages of development. Almost all studies were qualitative (with one using the Delphi method), and none specifically examined the effectiveness of CD practices. Qualitative research is crucial for deepening our understanding of how occupational therapists can contribute effectively to CD. However, practitioners and services often operate within institutional and remuneration frameworks that require demonstrable outcomes, meaning that quantitative evidence may also be needed. Addressing this need remains an ongoing challenge. The limited number of quantitative studies may be linked to one of the key findings of this review: ‘the ambiguity surrounding CD outcome*s*’, which makes them challenging to reliably quantify. In addition, the potential outcomes of CD, such as social change, community ownership, and community participation, are too complex to measure. For example, to measure social change, it is necessary to define transformation theoretically as a measurable phenomenon and establish specific measurement methods (Garonna & Triacca, 1999). Alternatively, approaches such as social impact measurement, which utilises qualitative methods (e.g., semi‐structured interviews) to assess social outcomes (Studer, 2022; van der Westhuizen & Visagie, 2025), may offer a more appropriate means of evaluation in this context. This approach requires shifting focus to capturing changes using qualitative methods, rather than relying solely on objective indicators to measure results only. Regardless of the approach adopted, it should be aligned with the conclusions of broader debates on evidence generation in occupational therapy, particularly in the community of practice and socially complex contexts.

The concentration of research within a limited group of authors and countries is evident, reflecting the constrained contextual diversity among the identified articles. Several studies have been conducted by the same researchers or research teams (Hyett et al., 2017, 2019; Irvine‐Brown et al., 2022, 2021; Lauckner et al., 2007, 2011; Lauckner & Stadnyk, 2014; Leclair et al., 2016, 2019), indicating a relatively narrow contributor base. Moreover, there is a notable absence of studies from regions such as the United States, South America, and Asia. Given the ongoing calls to promote community practice globally within occupational therapy (World Federation of Occupational Therapists, 2016; World Federation of Occupational Therapists et al., 2017), research from these regions is essential to expand the relevance and applicability of knowledge surrounding CD practice. This absence may, in part, reflect limitations in the search strategy; specifically, the failure to capture contextually specific terminology that better reflects how CD is conceptualised and implemented in different regions. As reported by the International Association for Community Development, the term CD is used mainly in a limited number of countries, whereas other regions use different terms or labels (International Association for Community Development, 2015). For example, in some South American countries, forms of community action, although not referred to as CD, have been described in occupational therapy contexts (Bianchi & Malfitano, 2022). Future reviews may benefit from incorporating additional key terms, such as ‘social occupational therapy’ and ‘community participation’, which have been identified as related or overlapping concepts, to explore a more diverse range of CD‐related practices.

### 4.2 Occupational therapy perspectives on CD

Qualitative analysis further suggested that CD is highly compatible with occupational therapy as the two share common values and principles. At the same time, limitations in existing occupational therapy theories and conventional classifications of occupations were noted, indicating a need for more advanced perspectives that extend beyond traditional occupational therapy paradigms, such as the application of an occupational justice lens and a focus on collective occupation within communities. The analysis also highlighted the specific skills required for CD practice, such as understanding communities, power sharing, and relationship building.

As it requires a holistic perspective that transcends the conventional individualising biomedical paradigm, CD is conceptually aligned with emerging practical fields such as social occupational therapy (Malfitano et al., 2014) and social transformation (Schiller et al., 2023). These fields have increasingly been discussed through the lens of ‘communities of practice’ in occupational therapy (Hyett et al., 2023). However, knowledge within these domains often remains embedded in the tacit and context‐dependent experiences of individual practitioners, limiting its transferability and cumulative development. To advance CD as a legitimate and sustainable area of occupational therapy, fostering intentional mechanisms of knowledge exchange and critical dialogue among practitioners is essential. Doing so will not only support the dissemination of practical insights but also contribute to the theoretical maturation of this field within the profession.

Although interest in CD is growing, as evidenced by our content analysis, there remains a lack of knowledge, educational systems, and institutional support for promoting CD practice implementation. As our findings illustrate, similar discussions have persisted for decades (Hoerder et al., 2025; Lauckner et al., 2007), suggesting that critical issues related to CD remain unresolved. In some countries, CD or similar practices (e.g., social occupational therapy) have been incorporated into university curriculums (Galvaan et al., 2022; Irvine‐Brown et al., 2020) and fieldwork training (Mthembu et al., 2017; Vermeulen, 2015). However, recent studies focusing on occupational therapists engaged in CD practice have repeatedly reported practitioners' lack of confidence, as well as uncertainty and ambiguity regarding the scope and content of their work (Hoerder et al., 2025; Irvine‐Brown et al., 2021; Lauckner et al., 2019). These findings suggest that education in training institutions and practice‐based support for CD remain limited or underdeveloped in many countries. To address these challenges, it is essential to prioritise the enablers identified in this review, such as the development of theoretical knowledge and empirical evidence, the establishment of comprehensive educational systems, and the institutional promotion of CD practice. In particular, the inclusion of dedicated CD‐focussed units that address what CD practice entails and how it can be integrated into occupational therapy practice is needed in pre‐service occupational therapy training. Strengthening such educational foundations would contribute to more effective CD practice delivered by occupational therapists.

The interpretation of CD in occupational therapy tends to emphasise power sharing and critical consciousness. This trend aligns with Freire's (1972) philosophy, which has been explicitly referenced in several articles (Irvine‐Brown et al., 2022; Townsend et al., 2007; Windley, 2011). In the broader field of community organisation for social change, Freire is often contrasted with Saul Alinsky (Martinson et al., 2022). Whereas Alinsky's practical ideology emphasises the mobilisation of communities through leadership by organisers to confront and transform power structures, Freire's approach focuses on developing a critical consciousness among individuals through dialogue and mutual learning. The Freirean orientation observed in how CD is conceptualised in occupational therapy may reflect the profession's foundational emphasis on client‐centredness and collaboration. This suggests that occupational therapists may be particularly well positioned to adopt CD practices grounded in empowerment and participatory engagement.

### 4.3 Limitations

This scoping review collected and analysed the literature on CD in the field of occupational therapy, covering a wide range of topics. However, several limitations should be acknowledged. First, although the findings provide a comprehensive perspective, the literature was selected based on its prototypical use of the terms ‘community development’ and ‘community‐centred practice’. As a result of this study, this area is also discussed within the concept of community of practice, and terms such as ‘social occupational therapy’ and ‘community participation’ were identified as related or overlapping concepts. However, these terms were not used as primary search terms in this review, potentially limiting the comprehensiveness of the search. Furthermore, several articles without a detailed review of their content were excluded from the target literature, either because they could not be obtained or because of the language in which they were written. Moreover, this review relied on a comprehensive set of databases accessible through the authors' institution, which might have restricted access to a limited number of relevant journals (e.g., those searchable only via Scopus), nevertheless. Therefore, the articles identified in the current study may not fully capture the breadth of this field.

Second, this review conducted a qualitative analysis of how CD was described in the included articles and inductively identified thematic categories. Although this resulted in a comprehensive perspective that focussed on a broad range of topics, it did not allow for a detailed discussion and analysis of each topic. Further reviews and research are warranted to focus on some of the topics identified to develop this area. Focusing on the category of ‘barriers and enablers to engaging in CD practice’ and examining the current progress in the area may help to identify new challenges and enablers and barriers that can be managed in CD practice.

Third, this review did not fully address a fundamental tension between CD and traditional occupational therapy practice. CD does not rely on a therapeutic relationship with an individual client, and its focus is inherently collective and community‐driven. In contrast, occupational therapy has historically been grounded in individualistic and therapeutic approaches, and as noted by CD scholars (Irvine‐Brown et al., 2021; Lauckner, 2010; Lauckner et al., 2012; Lauckner & Stadnyk, 2014; Leclair, 2010; Leclair et al., 2019), there are limitations to applying existing occupational therapy theories directly to CD practice. These differences suggest that the two orientations may reflect distinct underlying paradigms and bringing them together requires more than simply adopting CD as an additional approach.

Relatedly, although the present review identified issues such as ‘traditional authoritarian or medically oriented perspectives’ at the individual level, it was beyond the scope of this study to analyse the deeper conceptual contradictions between occupational therapy and CD. Future research is urgently needed to examine how occupational therapists can navigate and reconcile these paradigms and to explore what kinds of theoretical, educational, and practice frameworks are required for occupational therapists to engage in CD in a way that is both effective and appropriate. For instance, although Lauckner et al. (2019) conceptualised CD practice as a multi‐layered practice and proposed focusing on this continuum of practice, there remains limited understanding of how occupational therapists can shift their perspectives from the individual level to the community or social level. Future studies focusing on this issue are needed to enhance occupational therapists' understanding of CD.

Finally, although several definitions of CD, both occupational therapy based and non‐occupational therapy based, were identified in the included articles, this review did not examine how these definitions were used or the extent of their acceptance. The review therefore provides only an overview of the definitions reported in the literature. Further detailed analyses of how these definitions are applied, such as through concept analysis (e.g., Rodgers, 2000), are required to develop deeper insights into CD in occupational therapy.

### 4.4 Implications for practice and research

This review provides comprehensive information regarding CD in occupational therapy using inductive content analysis. By combining the analysis results of the research trends in this field, the identified topics can guide practitioners, educators, researchers, and policymakers in developing and promoting occupational therapist engagement in CD.

The results underscore the urgent need for the development of theoretical frameworks and empirical evidence supporting CD practice as well as the establishment of educational and institutional systems that can facilitate its implementation. Educational systems targeting students and current practitioners should be prioritised to foster confidence and clarity in engaging with CD. The topics identified in this review may serve as a foundation for designing such educational initiatives by providing themes needed to prepare for CD practice.

This review highlights the need for further attention to evaluating the outcomes of CD practices, developing guiding theoretical models, and re‐examining how occupations are classified, especially in ways that account for collective or shared occupations beyond individual occupations (Kantartzis & Molineux, 2017). Future research should address these issues to deepen the understanding and applicability of CD in occupational therapy.

In addition to educational and research priorities, organisational and institutional support are essential to promote CD through occupational therapists. It is important to respond to the need for institutional and financial support as indicated by the results of this study. For example, enabling CD‐related activities within existing workplace structures or creating accessible participation opportunities for interested practitioners could foster engagement. Moreover, support systems for those already involved in CD, such as communities of practice, opportunities to share experiences, and continued education, should be strengthened to sustain and expand this area of practice.

## 5 CONCLUSIONS

Occupational therapists discuss CD from distinct professional perspectives and values, with alignment between the core principles of CD and the philosophy of occupational therapy. However, several challenges persist, including limited research, insufficient educational infrastructure, lack of guiding occupational therapy theories, and institutional barriers. To advance this field, future studies should explore CD practices in diverse geographical and cultural contexts, develop theoretical frameworks to guide occupational therapists, and apply the concept of collective occupation to better capture the nature of CD practice. Strengthening educational systems and institutional support for both practitioners and students is also essential to promote sustained engagement with CD in occupational therapy.

## AUTHOR CONTRIBUTIONS

All authors meet the criteria for authorship. **Tetsuya Anzai:** Conceptualisation (lead); data curation (lead); formal analysis (lead); investigation (lead); methodology (equal); project administration (lead); visualisation (lead); writing—original draft (lead); writing—review and editing (equal). **Atsushi Kawabata:** Data curation (supporting); formal analysis (supporting); validation (lead); writing—review and editing (equal). **Norikazu Kobayashi:** Supervision (supporting); validation (supporting); writing—review and editing (equal). **Peter Bontje:** Conceptualisation (supporting); formal analysis (supporting); investigation; methodology (equal); supervision (lead); validation (supporting); project administration (supporting); writing—review and editing (equal). All authors approved the final version for publication and agreed to be accountable for all aspects of the work. Tetsuya Anzai led all stages of the review as this work formed part of his doctoral research.

## CONFLICT OF INTEREST STATEMENT

The authors declare no potential conflicts of interest with respect to the research, authorship, or publication of this article.

## Data Availability

Research data are not shared.
